# Psychometric testing of the 20-item Self-Management Assessment Scale in people with chronic obstructive pulmonary disease

**DOI:** 10.1038/s41598-025-31228-2

**Published:** 2025-12-11

**Authors:** Sara Lundell, Senada Hajdarevic, Anna-Britt Coe, Ulf Isaksson

**Affiliations:** 1 Department of Sociology, Umeå University, Umeå, Sweden; 2 Department of Nursing, Umeå University, Umeå, Sweden; 3 Department of Public Health and Clinical Medicine, Family Medicine, Umeå University, Umeå, Sweden; 4 Department of Social and Psychological Studies, Karlstad University, Karlstad, Sweden; 5 Present Address: Department of Community Medicine and Rehabilitation, Umeå University, Umeå, Sweden

**Keywords:** Chronic disease, COPD, Psychometry, Reliability, Self-management support, Validity, Rehabilitation, Chronic obstructive pulmonary disease, Lifestyle modification, Health care, Patient education

## Abstract

Chronic obstructive pulmonary disease (COPD) represents a public health challenge and self-management support is crucial to prevent deterioration of health. The Self-Management Assessment Scale was developed to screen for prerequisites for self-management but has not been tested on people with COPD. The study aimed to evaluate the psychometric properties and internal structure of the 20-item Self-Management Assessment Scale in a Swedish COPD population. The 20-item Self-Management Assessment Scale was tested in a sample of 173 participants with a verified COPD diagnosis. Data collection was performed between September 2021 and September 2022. Assessment of content validity by an expert group, confirmatory factor analysis, and test-retest reliability were performed. The scale content validity index (S-CVI) was 0.87, and the number of missing items was low. A moderate to good goodness-of-fit of a five-factor solution was shown. The five factors, Knowledge, Goals for the future, Daily routines, Wellbeing, and Social Support, had correlations within the range of 0.59 to 0.89, with the strongest correlation between Wellbeing and Daily routines. The internal reliability was high for the instrument (0.93) and for each factor (0.73–0.85). Test-retest reliability was good, with no significant difference between scale or factor scores. Good validity and reliability were shown for the Self-Management Assessment Scale and its five key domains. The low number of missing items indicates that the instrument is easy and quick for people to complete. The Self-Management Assessment Scale is, therefore, considered a valuable tool in clinical practice.

## Introduction

Self-management is a crucial healthcare intervention worldwide. People who are ill often face the need to manage their symptoms and illnesses on their own. Moreover, self-management is increasingly linked to shared understandings of what constitutes good citizenship by acting as independent persons and avoiding burdening the healthcare system unnecessarily. Self-management is often used by people at home, aiming to prevent illness and stay healthy^1^. Barlow et al.^2^ define self-management as an individual’s ability to manage symptoms, treatment, physical and psychological consequences, and lifestyle inherent in living with a chronic condition and as a dynamic and continuous self-regulating process for maintaining a satisfactory quality of life. For people with chronic illness, self-management is important in helping them preserve physical capacity and experience wellbeing^3^. Indeed, it is suggested to be a precursor of patient empowerment and improved patient outcomes^4^. However, for some people, self-management can be a struggle and burden in their everyday lives^5^, which may negatively impact people and their quality of life.

Chronic obstructive pulmonary disease (COPD) represents a public health challenge and is a significant cause of morbidity and mortality worldwide^6^. The disease generates substantial costs for society and the healthcare system, where the highest costs are related to hospitalizations and pharmacological treatment^7^. Self-management interventions can prevent deterioration of health and subsequent hospitalizations and improve quality of life^8^. Given limited healthcare resources, supporting self-management among people with COPD is crucial both for their health and the health system. Self-management interventions are recommended in treatment guidelines for COPD^6^. However, more knowledge is needed about prerequisites for self-management and how these can be fostered to promote self-management effectively. Instruments for measuring the need for self-management support are missing in COPD care but can be found for other conditions. Few previous instruments for assessing self-management have been developed with the specific aim of guiding self-management support^9^. The Self-Management Assessment Scale (SMASc) was initially developed in Swedish to screen for prerequisites for self-management among people with diabetes, based on the research by Jutterström^10^ and Hörnsten et al.^11^. Specifically, diabetes nurses could use this scale to better understand the needs and challenges of people with diabetes, especially those with Type-2, and to support them in actively managing their disease. An initial instrument containing ten items was developed and psychometric tested by Öberg et al.^12^. The items were categorized into five domains, with two items per domain. The instrument has been appreciated by diabetes nurses, who felt that the conversations with patients were redirected from giving information and knowledge about the illness towards a more holistic view of the patient’s entire life situation^13^. When the instrument was implemented in clinical practice, the team who developed the instrument received indications that certain items may have been difficult to interpret and that some items were too similar. In addition, since an instrument should contain at least three items per domain to be psychometrically sound^14,15^, the developers decided to add one item per domain in the first part (A), resulting in a total of 15 items covering a broader perspective of each domain. In connection with this, the developers also gathered information from caregivers and patients regarding their perceptions of the various questions and reformulated certain items. In addition, to make the instrument more person-centered, a new part (B) with five questions was added about how important each domain was to the patients personally. This decision was based on the transtheoretical therapy^16^, person-centered practice^17^, and motivational interviewing^18^. These items could facilitate discussions about the patients’ motivation for behavior change in self-management, related to each domain. We argue that the revised instrument does not only assess needs but also the prerequisites individuals have to base their self-management on. Therefore, the instrument currently contains 15 items that assess the person’s prerequisites and five items where the person assesses how important each domain is to them. However, this version has not been psychometrically tested. Moreover, none of the two SMASc versions have previously been tested on people with COPD.

### Aim

The study aimed to evaluate the psychometric properties and internal structure of the 20-item SMASc in a Swedish COPD population.

## Methods

### Questionnaire

A paper questionnaire consisting of questions about demographic background, COPD-related information, and the SMASc was used to collect data.

The SMASc consists of two parts. The first part (A) consists of 15 items and assesses the person’s prerequisites for self-management in chronic conditions, and the second part (B) assesses the personal importance of each area. The combined score (A + B) reflects the need for self-management support in relation to chronic condition. The instrument assesses five key domains: Knowledge, Goals for the future, Daily routines, Wellbeing and Social support.

The first domain assesses Knowledge, which pertains to facts and information within the realm of informational health literacy, something Batterham et al.^19^ and Sørensen et al.^20^ note plays a critical role for health literacy. For instance, it evaluates the need for additional information related to various aspects such as diseases, physiological processes, medications, dietary choices, exercise, or strategies for managing conditions. Moreover, knowledge needs to include access to contact details for healthcare facilities and professionals and connections with social networks, patients, and voluntary organizations. These are all essential elements of knowledge that individuals dealing with chronic conditions may require.

The second domain, Goals for the future, encompasses one’s objectives and strategies for daily life. Having specific, concrete goals for self-management activities is more advantageous than vague, unspecified plans. The absence of any plans or even the motivation to formulate a plan may indicate a lack of readiness for change, as highlighted by Bratzke et al.^21^, Fitzpatrick et al.^22^, and Hoffmann et al.^23^. Hajdarevic et al.^24^ indicate that prospective intentions are interconnected with one’s coping mechanisms. These goals also encompass the necessity to seamlessly integrate illness management into one’s daily life, as exemplified by practical activities like self-monitoring, medication adherence, and adjustments in diet and exercise, if required, as mentioned by Jutterström^10^.

The third domain, Daily routines, is concerned with effectively managing one’s condition in daily life. This encompasses establishing consistent patterns related to exercise and dietary choices. It also involves being prepared for changes during travel or when facing new circumstances. The mere recognition of the need for altered daily routines is typically insufficient to instigate behavioral change, as highlighted by Jutterström et al.^25^. One approach to creating and maintaining daily routines, which can enhance self-efficacy and the seamless integration of illness management, is seeking guidance from healthcare professionals such as nurses, dietitians, or physiotherapists, as suggested by Abubakari et al.^26^, as well as Aljasem et al.^27^. This collaborative effort can contribute to strengthening one’s ability to manage their condition and effectively incorporate it into their daily life, as supported by Boström^28^, Hernandez^29^, and Jutterström^10^.

The fourth domain, Wellbeing, revolves around the emotional and existential journey individuals diagnosed with a chronic conditions undergo to attain a sense of normalcy and contentment, as Jutterström^10^ emphasized. This process of emotional adjustment often entails reevaluating one’s identity and roles. It also involves grappling with challenges like the apprehension of complications or even the specter of death that can be associated with long-term conditions. When emotions are dominated by fear, as opposed to a sense of life satisfaction, it can impede rational decision-making concerning self-management. Conversely, an insufficient emotional response can hinder effective self-management, as Hörnsten et al.^11^ noted.

The last dimension, Social Support, focuses on enhancing one’s social connections to facilitate self-management, as Fivecoat et al.^30^ and Hasan et al.^31^ observed. This strengthening of social networks can encompass involvement from family members, friends, and colleagues at various levels. Additionally, patient organizations and other voluntary groups play a vital role in this context. It is worth noting that family members and social circumstances can sometimes present challenges to effective self-management, as Koetsenruijter et al.^32^. Furthermore, as highlighted in various studies, healthcare professionals can either provide support or potentially hinder self-management and the integration of illness into one’s life^33,34^.

### The scoring of SMASc

#### Scoring part A

The assessment employs a six-point Likert scale, ranging from 1 (Strongly disagree) to 6 (Totally agree), for scoring each item. A mean score is computed for part A and for each domain as follows: Knowledge: ((A1 + A4 + A13)/3), Goals for the future: ((A5 + A9 + A14)/3), Daily routines: ((A2 + A7 + A10)/3), Wellbeing: ((A3 + A6 + A12)/3) and Social support: ((A8 + A11 + A15)/3) where each domain in part A gets a total score between 1 and 6.

#### Scoring part A + B

All response options in subscale B are reversed, ranging from 1 (Not at all important) to 6 (Extremely important). An index is computed for each subscale by adding the respective mean score from part A (prerequisites for self-management) to the corresponding question score from subscale B (importance of the area). For Knowledge, item B3 is added; for Goals for the future, item B5 is added; for Daily routines, item B4 is added; for Wellbeing, item B2 is added; and finally, for Social support, item B1 is added. Each domain in part A + B gets a total score between 2 and 12.

#### Interpretation of the scores for part A + B

A score of 2–4 is interpreted as indicating a need for self-management support, while a score of 9–12 signifies no need for self-management support. A score of 5–8 suggests that there is something to follow up on, and together with the patient, discuss and support their decision-making.

Within each domain, a lower score indicates a greater requirement for self-management support. It is important to note that this instrument does not calculate an overall self-management assessment score.

### Content validity

The content validity of each item in the Swedish version of the questionnaire was rated by an expert panel, according to guidelines^35^. The panel consisted of two professors, one associate professor, and two PhD researchers. All have extensive experience working with people with COPD in clinical settings and in physiotherapy, medicine, nutrition, and nursing research. Swedish was four experts’ first language, and the fifth had Norwegian as the first language. The experts rated the relevance of each item related to the concept of self-management using a scale from 1 to 4: 1 = not relevant, 2 = less relevant/unable to assess the relevance, 3 = quite relevant, or 4 = very relevant and succinct. They were asked to suggest potential changes to the item’s wording and to evaluate whether the introductory text and instructions were clear or if any changes were needed. As a result, a short description of self-management was added to the introduction, and the wording of several items was changed to make them easier to understand. After the revision, the expert group made a second content validity assessment.

### Participants

The revised version of the 20-item SMASc was tested in a sample of people with a verified COPD diagnosis according to the Global Initiative for Chronic Obstructive Lung Disease (GOLD)^6^. Several recruitment strategies were used during the recruitment period (September 2021 to September 2022). First, primary and specialist care professionals in eight of Sweden’s 21 healthcare regions recruited participants among their patients. Second, participants were recruited through advertisements on webpages about COPD, social media groups, email newsletters, and lectures directed to people with pulmonary diseases. A smaller sample (*n* = 31) of participants was asked to complete the questionnaire a second time after 2–3 weeks to enable a test-retest analysis, which 26 participants answered. Participants took the paper questionnaire with them to fill it in on their own.

In total, 320 individuals were asked to participate in the study, and 58% (*n* = 185) completed and submitted the questionnaire. Eleven participants were excluded since no COPD diagnosis, according to GOLD^6^, could be verified, and one was excluded since a signed consent form was missing. Finally, 173 participants were included in the study, resulting in a subject-to-item ratio of 11.5:1, within the range of acceptability according to Costello and Osborne^36^, and 26 were included in the test-retest analysis. These participants were the same as in another study^37^. The participants included are presented in detail in Table 1.

**Table 1:** Background data of the included participants.

Characteristics	Total, *n* = 173	Test-retest, *n* = 26
*Sex (* *n* * = 173)*		
Women, n (%)	118 (68)	17 (65)
Men, n (%)	55 (32)	9 (35)
Age (*n* = 172), mean ± SD	74.0 ± 7.99	74.6 ± 8.30
FEV_1_, % predicted (*n* = 171), mean ± SD (min-max)	51 ± 19 (14–107)	48 ± 17 (24–107)^1^
FEV_1_/FVC, % (*n* = 173), mean ± SD (min-max)	48 ± 12 (18–69)	48 ± 11 (18–66)^1^
CAT (*n* = 171), points, mean (SD)^2^	16.6 ± 7.69	19.4 ± 6.22
Length of diagnosis (*n* = 156), year, mean (SD)	11.3 ± 7.71	11.5 ± 7.68
Living alone (*n* = 169), n (%)	73 (42)	10 (38)
*Level of education (n* *= 173)*		
Primary school, n (%)	71 (41)	9 (35)
Upper secondary school, n (%)	65 (38)	8 (31)
Tertiary education, n (%)	37 (21)	9 (35)

^1^One missing ^2^Higher number indicates increased symptoms.

CAT, COPD assessment test; COPD, Chronic obstructive pulmonary disease; FEV1, forced expiratory volume in one second; FVC, forced vital capacity; SD, Standard deviation.

### Ethical approval and consent to participate

The study was performed in line with the principles of the Declaration of Helsinki. All participants received written information about the study aims, voluntary participation and assured confidentiality. Written informed consent was received from all participants. The Swedish Ethical Review Authority approved the study, DNr 2021–02367.

**Table 2:** Updated content validity, median and distribution in percent for each item and response alternatives of the Self-Management assessment Scale, SMASc (*n* = 173).

	Items	Median	1	2	3	4	5	6	Missing*	I-CVI
A1	I have sufficient knowledge about my illness *Jag har tillräcklig kunskap om min sjukdom*	4	4.0	6.9	22.5	19.1	34.7	12.1	0.6	1.0
A2	I have created daily routines to manage my illness *Jag har hittat dagliga rutiner för att hantera mitt sjukdomstillstånd*	5	2.3	5.2	16.8	25.4	27.2	23.1	0.0	1.0
A3	I find joy in everyday life despite my illness *Jag finner glädje i vardagen trots min sjukdom*	5	1.7	2.9	11.6	20.2	38.2	25.4	0.0	0.6
A4	I have received sufficient information about my medical condition *Jag har fått information om mitt sjukdomstillstånd i tillräcklig utsträckning*	5	4.0	7.5	15.0	19.7	29.5	24.3	0.0	0.6
A5	I have thoughts and ideas on how to manage my illness in the future *Jag har tankar och idéer om hur jag kan hantera min sjukdom i framtiden*	4	7.5	14.5	22.5	24.3	19.7	11.0	0.6	1.0
A6	I can emotionally cope with living with my illness *Jag orkar känslomässigt med att leva med min sjukdom*	5	0.6	8.1	11.0	25.4	32.9	21.4	0.6	1.0
A7	I know how to deal with my illness in everyday life *Jag vet hur jag skall hantera sjukdomen i vardagen*	5	1.7	3.5	16.2	27.2	27.7	22.5	1.2	1.0
A8	I have people who support me to make self-management work *Jag har dem som stöttar mig för att egenvården ska fungera*	5	6.9	9.2	15.0	14.5	26.6	26.6	1.2	0.8
A9	I am confident that I can influence my future health *Jag hyser tilltro till att jag kan påverka min framtida hälsa*	4	3.5	12.1	19.1	22.5	27.2	15.0	0.6	0.8
A10	I have habits that are appropriate for living with my illness *Jag har vanor som är lämpliga för att leva med min sjukdom*	4	3.5	5.2	15.6	33.5	28.9	12.7	0.6	0.8
A11	My family and friends help me take care of my illness *Mina närstående hjälper mig att ta hand om min sjukdom*	4	13.9	11.6	13.3	19.1	18.5	22.0	1.7	0.4
A12	I feel satisfied with my situation *Jag känner mig tillfreds med min situation*	4	11.6	9.2	19.7	20.2	24.9	13.9	0.6	0.8
A13	I understand how my illness can develop *Jag förstår hur min sjukdom kan utvecklas*	5	3.5	4.0	11.0	19.1	34.7	26.6	1.2	0.8
A14	I have concrete plans on how to deal with my illness *Jag har konkreta planer för hur jag ska hantera min sjukdom*	4	7.5	15.6	26.6	19.1	19.7	11.6	0.0	1.0
A15	I have good social support which makes it easier for me to cope with my illness on a daily basis. *Jag har ett gott socialt stöd vilket underlättar för mig att klara vardagen med min sjukdom*	5	2.9	11.6	15.6	17.9	27.7	23.7	0.6	0.8
B1	How important is it for you to have support to manage your illness? *Hur viktigt är det för dig att ha stöd för att hantera din sjukdom?*	5	4.0	6.9	11.0	19.1	27.2	31.2	0.6	1.0
B2	How important is it for you to be emotionally balanced in order to manage your illness? *Hur viktigt är det för dig att du känslomässigt är i balans för att kunna hantera din sjukdom?*	5	2.9	3.5	5.8	20.8	31.2	35.3	0.6	1.0
B3	How important is it for you to have good knowledge about your illness? *Hur viktigt är det för dig att ha goda kunskaper om din sjukdom?*	5	0.0	1.2	1.7	16.8	34.7	44.5	1.2	1.0
B4	How important is it for you to have daily routines to manage your illness? *Hur viktigt är det för dig att ha dagliga rutiner för att hantera din sjukdom?*	5	1.2	1.2	8.7	18.5	37.6	32.4	0.6	1.0
B5	How important is it for you to have goals for your future health? *Hur viktigt är det för dig att du har mål för din framtida hälsa?*	5	1.7	2.3	8.7	22.0	31.8	32.9	0.6	1.0
									**S-CVI**	**0.87**

The English version is in plain text, and the Swedish version is in italics.

*Percentage of internal missing items.

### Statistical analysis

The item (I-CVI) and scale (S-CVI) content validity index^38^ were based on the expert group assessment, where S-CVI was calculated as the experts’ mean agreement for the whole scale.

The distributional properties for each item were assessed in percentages. Five participants gave two answers on one item each, i.e., for 0.1% of all items. This was solved by flipping a coin to see which answer should be used in the analysis.

A confirmatory factor analysis was conducted for part A to evaluate the goodness of fit using various statistical indicators, including the chi-square (χ2) and chi-square divided by degrees of freedom (χ2/df), the Tucker–Lewis index (TLI), Standardized Root Mean Squared Residual (SRMR), and the root mean square error of approximation (RMSEA). These indices were interpreted using established cut-off values: TLI > 0.95, SRMR < 0.08, and RMSEA < 0.06, indicating good model fit^39,40^. The internal consistency of the data was assessed by calculating Cronbach’s alpha. To evaluate the assumption of multivariate normality prior to conducting the confirmatory factor analysis (CFA), Mardia’s test was performed. The results indicated p-values < 0.001, suggesting that there were significant deviations in skewness/kurtosis. Given these findings, robust estimation methods were applied to account for potential non-normality. This step ensured that the CFA results were based on appropriate statistical assumptions, thereby contributing to the overall validity of the model.

Wilcoxon rank was used to evaluate the stability of item response and for the factors over time, i.e., test-retest reliability, since the data were not normally distributed (Shapiro-Wilk test). Jamovi 2.2.5 and IBM SPSS Statistics 28 were used as statistical software.

## Results

### Content validity

The I-CVI from the second assessment of the expert group varied between 0.4 and 1.0 for the twenty items in SMASc, and the S-CVI was 0.87 (Table 2).

### Distributional properties

The response distribution for each item is presented in Table 2. The number of missing items was low for all items (0.0–1.7.0.7%), with four items having no missing answers. A ceiling effect, i.e., ≥ 15% of the responses in the highest point in the scale, was observed for all items except A1, A5, A10, A12, and A14.

### Confirmatory factor analysis

**Fig. 1:**
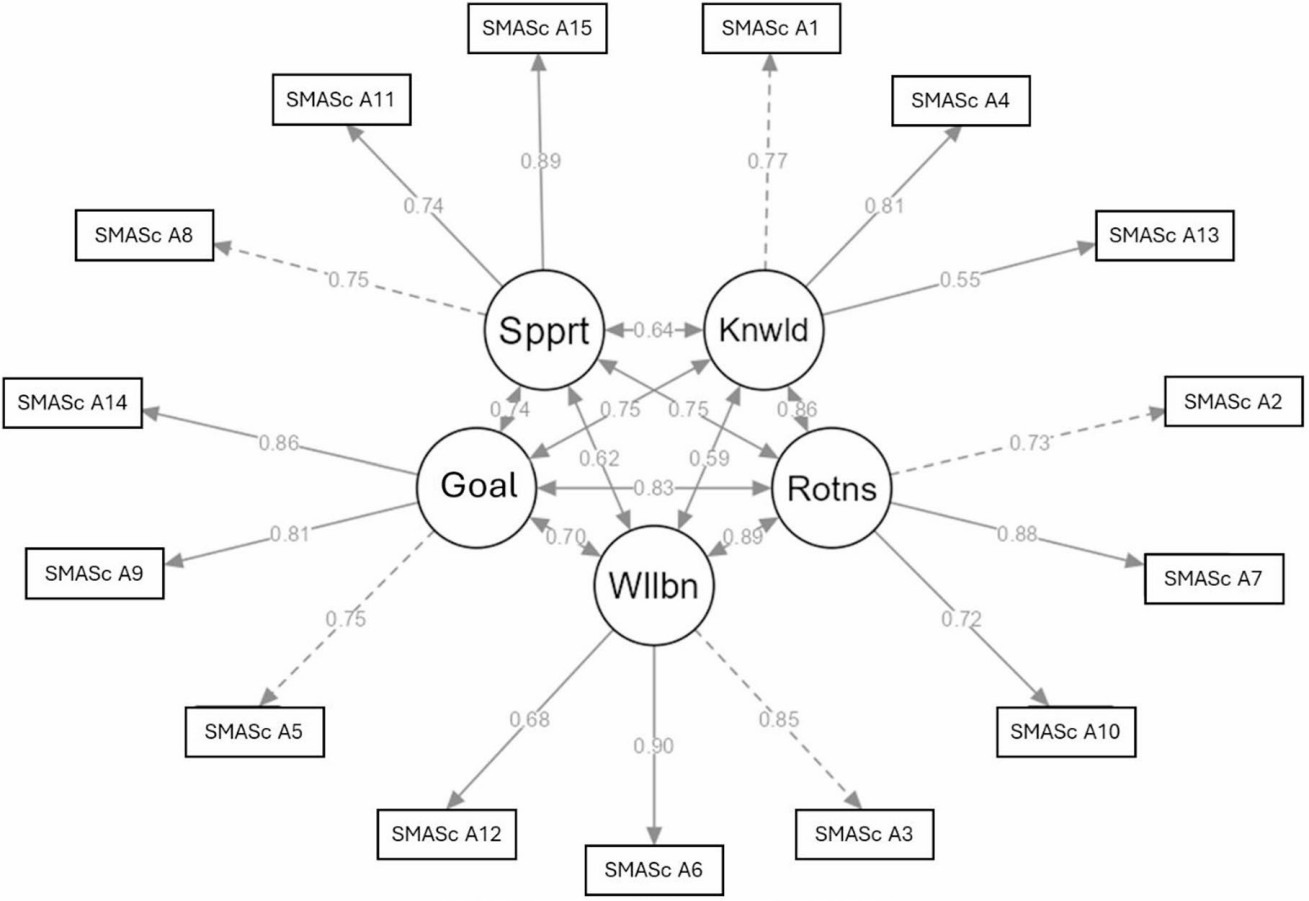
Model of part A from the 20-item self-management assessment scale with standardized factor loadings. Note: Dotted lines indicate reference indicators for latent variables. In this standardized model, all factor loadings—including reference indicators—are freely estimated. Abbreviations: Spprt = Social support, Knwld = Knowledge, Goal = Goals for the future, Wllbn = Wellbeing, Rotns = Daily routines.

A confirmatory factor analysis was performed to test the goodness-of-fit of the five-factor solution. Chi-square was significant (χ2 = 184.93, df = 80, *p* < 0.001). However, the relative chi-square (χ2/df) was 2.31. SRMR was 0.063, TLI was 0,869, and RMSEA was 0.110, indicating a moderate to good goodness-of‐fit. The final model, which includes path coefficients, is shown in Fig. 1. The correlations among the factors fell within the range of 0.59 to 0.89, with the strongest correlation between Wellbeing and Daily routines.

### Internal reliability

The internal consistency of each domain was assessed using Cronbach’s alpha, yielding the following values: Knowledge (α = 0.73), Goals for the future (α = 0.84), Daily routines (α = 0.81), Wellbeing (α = 0.83), and Social support (α = 0.85). The overall Cronbach’s alpha for the entire instrument was calculated to be 0.93.

### Test-retest reliability

In the test-retest analysis, no significant difference was shown between scale scores at baseline and after 2–3 weeks, neither for the items nor for the factors (Table 3). The absolute agreement for the items was between 27% and 69%.

**Table 3:** Test-retest reliability for the items and factors in SMASc (*n* = 26).

Item	Test, mean (SD)	Retest, mean (SD)	*p*-value	e%
A1	3.77 (1.18)	3.73 (1.19)	0.857	42%
A2	4.20 (1.16)	5.30 (1.08)	1.000	42%
A3	4.69 (0.97)	4.54 (1.30)	0.566	62%
A4	4.08 (1.29)	3.96 (1.10)	0.559	58%
A5	3.46 (1.17)	3.35 (1.20)	0.713	35%
A6	4.12 (1.11)	4.12 (1.18)	1.000	54%
A7	4.00 (0.98)	4.15 (1.19)	0.444	54%
A8	4.12 (1.27)	3.96 (1.46)	0.444	38%
A9	3.77 (1.14)	3.92 (1.20)	0.444	58%
A10	4.08 (0.89)	3.88 (0.99)	0.259	46%
A11	3.68 (1.40)	3.92 (1.22)	0.282	35%
A12	3.23 (1.66)	3.38 (1.36)	0.557	27%
A13	4.35 (1.20)	4.46 (1.33)	0.677	42%
A14	3.31 (1.19)	3.27 (1.25)	0.814	46%
A15	4.08 (1.26)	4.20 (1.19)	0.559	35%
B1	4.12 (1.14)	4.54 (1.14)	0.061	50%
B2	4.69 (0.97)	4.69 (1.05)	1.000	54%
B3	5.12 (0.71)	5.04 (0.92)	0.490	69%
B4	4.58 (1.17)	4.54 (1.21)	0.802	54%
B5	4.54 (0.96)	4.62 (1.20)	0.691	54%
Factor 1 *Knowledge*	5.92 (0.94)	5.97 (0.82)	0.696	N/A
Factor 2 *Everyday routines*	6.47 (1.39)	6.57 (1.35)	0.526	N/A
Factor 3 *Wellbeing*	6.32 (1.54)	6.32 (1.39)	0.845	N/A
Factor 4 *Goals for the future*	5.97 (1.03)	5.90 (1.17)	0.741	N/A
Factor 5 *Social Support*	6.64 (1.03)	6.26 (1.26)	0.331	N/A

Abbreviations: e% = exact agreement, SD = standard deviation.

## Discussion

The main finding in this study indicates that the 20-item version of the Self-management Assessment Scale (SMASc) is psychometrically sound. As presented in a previous study^12^, the initial version of the instrument was grounded in theory regarding the integration of illness, the viewpoints of patients on self-management, and the concept of person-centered care in Type 2 Diabetes. However, that version consisted of only ten items and was therefore not fully psychometrically sound, which we have now remedied by adding one item to each domain. In addition, a new part (B) have been added with questions about the personal importance of each domain. By combining these part, a combined score reflecting the need for self-management in relation to chronic conditions can be assessed. In developing the instrument, we also saw an opportunity to test the instrument further on people with another chronic disease, namely COPD, as we believe the instrument can be more generic. Successful self-management interventions consist of several components, which are based on the patients’ needs and include follow-up with individualized feedback^41^. However, in previous interventions targeting COPD self-management, only few interventions were individualized based on the needs of the participants, and goal setting, emotional management and shared decision-making were rarely part of the interventions^42^. Thus, developing tools that help healthcare professionals identify patients’ needs is necessary to adapt a supportive approach and improve healthcare professionals’ ability to provide self-management support^4^.

The instrument’s content validity was assessed by experts as high (S-CVI = 0.87), close to excellent^38^. However, one of the items, item 11, had an I-CVI of only 0.4 since two experts (a physician and a nurse) rated the item very high. In comparison, three experts (two physiotherapists and a dietician) rated it low. It is difficult to explain this, but one reason may be that people have different perspectives depending on their profession. Another thing was that some experts commented that some items were considered too similar in wording. However, these questions are designed to triangulate a particular phenomenon, as suggested by Raubenheimer^14^ and Norman and Streiner^15^, and may therefore be perceived as similar. In addition, even though the instrument had very few missing values, item 11 stands out again, with 1.7% missing. Altogether, this indicates that item 11 may need to be revised and reworded slightly in further development of the instrument. When comparing item 11 with item 8, item 11 is about having people *helping* them with self-management, while item 8 is about having people *supporting* them with self-management. According to the Cambridge Dictionary^43^, there is a clear difference between giving help and support, where giving help is about doing part of the work yourself while giving support is about encouraging someone to succeed. This difference might be important to clarify when rewording the items. This is especially relevant for healthcare professionals’ awareness of their supportive capacity since promoting self-management needs to be based on trust, collaboration and active, sustained follow-up by skilled staff^4^.

The responses among the different items were relatively evenly distributed. However, several items had a ceiling effect (> 15%). In our opinion, this is a positive indication that many people with COPD can manage their self-management relatively well and independently. One of the ideas behind this instrument is to identify those needing support to become fully able self-manage their disease.

The confirmatory factor analysis indicated a moderate to good goodness-of-fit. The relative Chi-square was slightly higher than the original 10-item SMASc instrument (2.31 vs. 1.84) but still within the range of what is accepted as a good fit^40,44^. The model fit was slightly below the recommended levels for specific indices (RMSEA = 0.11; TLI = 0.87), indicating that the model does not fully capture the data structure. Possible reasons for this may be that certain relationships between indicators and latent factors are not optimally specified or that the sample size has affected the results. At the same time, SRMR was below the recommended 0.08^39^, indicating that the difference between the observed and expected correlations is small and therefore acceptable. Overall, the model provides some support for the theoretical structure; however, the results should be interpreted with caution. Future studies should consider testing alternative models or including more indicators. One explanation for the small discrepancy in goodness-of-fit between the original instrument and this, the extended instrument, is that people with Type 2 diabetes answered the original instrument^12^. In contrast, in this study, people with COPD were participants. It may be that the questions are interpreted slightly differently between different chronic diseases. Still, we nevertheless interpret that the instrument is more generic and should be able to be used among people with chronic conditions to assess the prerequisities, such as emotional and social, they have to base their self-management on. The internal reliability was also high, with Cronbach’s alpha for the different domains ranging from 0.73 to 0.84 and the alpha for the instrument as a whole being 0.93. This demonstrates high internal consistency among the items and indicates that they likely measure the same underlying construct or characteristic^45^. Reliability over time also showed that the instrument is stable.

Self-management, as the dominant form of care, intersects with the health system and healthcare professionals. It significantly impacts health outcomes by empowering and supporting people in acquiring knowledge, skills and resources to maintain their health^4,46^. Being asked about their personal circumstances is valued by patients, which can enhance collaboration between patients and healthcare provider^47^. However, when supporting people in self-management, healthcare professionals focus predominantly on informing and trying to educate^4,47,48^. Knowledge is necessary but not crucial for enhancing self-management and health outcomes, especially if it is the most significant part of given support^49,50^. Our study shows that wellbeing correlates with knowledge (*r* = 0.59), but social support, goals for the future and daily routines show even stronger correlations (*r* = 0.62–0.89), as presented in Fig. 1. We believe that all dimensions are important for improving a person’s wellbeing. For example, in adults with cystic fibrosis, decreased social support was shown to be associated with reduced physical and mental health^51^.

Furthermore, it seems important how self-management support is communicated and adapted^50^. Self-management can potentially enhance peoples’ health and wellbeing and is an essential part of person-centered care^4^. Person-centered care increases peoples’ ability to make informed decisions and creates an opportunity to avoid social and cultural iatrogenesis of self-medicalization^4,46^. However, previous research indicates that self-management interventions mainly focus on healthy lifestyle behaviors intended to maintain physical stability without focusing on psychological factors and higher-level skills such as symptom management behaviors^52^. Diabetes specialist nurses experienced that SMASc valuable in providing person-centered self-management support to people with type 2 diabetes in primary care since it addressed the patients’ personal needs^13^. Using person-centered support to build peoples’ capacities based on their health assets instead of mainly emphasizing information and technical skills is vital to improving people’s health^46,53^. Being a professional supporter of patient self-management activities means seeing patients as their own caregivers and experts on their condition with resources to achieve their desired behavior change instead of the often-defined biomedical goals. Creating such clinical partnership as a base for self-management support of patients, including their own defined goals, is necessary to further enhance patients’ competence, self-efficacy and capacity to self-manage their condition^48^. These aspects of partnership based on patient resources seem relevant not only for the identified relational effect between wellbeing and knowledge in this instrument but also for the relation between social support and goals for the future, as well as the relation between social support and daily routines and their further indirect regard to patient’s wellbeing.

### Methodological considerations

A strength with this study is that our sample was recruited from several parts of Sweden and is relatively similar to the COPD population in the national quality registry^54^. The registry includes a COPD population of nearly 70 000 patients from more than 1000 primary and secondary care clinics across Sweden. In the registry 44% are men (in our study 32%), the mean age is 70.3 years (in our study 74 years), have a mean FEV_1_% predicted of 60.3 (in our study 51), and a CAT score of 16.6 (in our study 13.4)^54^. In addition, during the development of the instrument, patient and healthcare representatives were involved in providing feedback on the items.

A limitation with this study is that no other questionnaire related to self-management was included to measure convergent validity. SMASc assesses prerequisites for self-management in chronic conditions (part A) and the need for self-management support in relation to chronic condition (part A + B). To our knowledge, no previous scale focuses on these aspects of self-management. Previous self-management instruments mainly focused on self-management behaviors, strategies and self-efficacy^9^.

Although some fit indices (e.g., TLI and RMSEA) did not reach the commonly recommended thresholds for good model fit, the model was specified based on strong theoretical and empirical foundations. Therefore, no post hoc modifications (such as correlating error terms or re-specifying the factor structure) were applied. This decision was made to preserve the conceptual integrity of the scale and ensure consistency with previous research^40,55,56^. As emphasized by Harrington^56^, model modifications should be guided by theory rather than solely by statistical suggestions.Future development and research.

SMASc has shown good reliability and validity for use among people with type 2 diabetes and people with COPD, indicating that it might be a more generic instrument. However, studies are needed about the use among other patient groups with chronic conditions. Furthermore, the instrument was developed in Swedish, and its psychometric properties have only been evaluated for the Swedish version in Swedish contexts. The English version presented in this manuscript is a translation suggested by the authors. However, the translation must be more thoroughly processed and psychometrically evaluated if the instrument should be used in English or any other language. In addition, a few adjustments in wording could be valuable in the Swedish version to provide better content validity, especially for item 11. During further development, it is important to consider including constructs such as readiness to act. In addition, studies comparing the correlation between SMASc and other self-management scales would be valuable. Finally, longitudinal research about needs for self-management support among people with COPD or other conditions, and its prediction of other outcomes such as quality of life, health behaviors or adherence is desired.

### Clinical implications

People with COPD can be viewed as a particularly vulnerable group. In a previous study they were considered a quiet group that rarely expressed requests from healthcare^57^. In addition, stigma has been stated as a core component of the lived experience of people with COPD, which can lead to delayed healthcare contacts, impaired self-management, and a reluctance to accept offered healthcare interventions^58^. This is especially important since it is known that people affected by long-term conditions such as COPD pass through a process of adaptation in which their self becomes affected and transformed. Therefore, healthcare professionals supporting these people in self-management need to expand their perspective beyond the biomedical understanding of a disease, and pay attention to this inner process in order to promote self-management^59^. Therefore, providing person-centered self-management support for people with COPD is crucial, and SMASc could be an important tool for healthcare professionals in this task.

## Conclusions

The Self-Management Assessment Scale (SMASc) showed a high internal consistency, a moderate to good goodness-of-fit, and close to excellent content validity. However, these results are limited to the Swedish version of the instrument, and the instrument could benefit from some revisions. The instrument is easy and quick for people to complete, as reflected in the low number of missing items. Based on these findings, we consider that SMASc might be a valuable tool in clinical practice. It can have the potential to identify peoples’ needs and facilitate discussions between healthcare professionals and patients about self-management strategies. Because of its five dimensions, the instrument can expand the healthcare professionals’ perspectives on COPD beyond biomedical understanding and give some directions concerning on what to focus on when supporting these patients in self-management. Consequently, the SMASc tool might improve person-centered support for people with COPD and other chronic conditions in clinical practice.

## Data Availability

The dataset supporting the conclusions of this article is available from the corresponding author, SL, upon reasonable request.

## Abbreviations

CAT: COPD assessment test
COPD: Chronic obstructive pulmonary disease
FEV_1_: Forced Expiratory Volume in one second
FVC: Forced Vital Capacity
GOLD: Global Initiative for Chronic Obstructive Lung Disease
I-CVI: Item Content Validity Index
RMSEA: Root Mean Square Error of Approximation
S-CVI: Scale Content Validity Index
SD: Standard deviation
SMASc: Self-Management Assessment Scale
SRMR: Standardized Root Mean Squared Residual
TLI: Tucker–Lewis index
